# The Role and Molecular Mechanism of Action of Surfactant Protein D in Innate Host Defense Against Influenza A Virus

**DOI:** 10.3389/fimmu.2018.01368

**Published:** 2018-06-13

**Authors:** I-Ni Hsieh, Xavier De Luna, Mitchell R. White, Kevan L. Hartshorn

**Affiliations:** Boston University School of Medicine, Boston, MA, United States

**Keywords:** surfactant protein D, influenza A virus, collectin, carbohydrate recognition domain, glycomics

## Abstract

Influenza A viruses (IAVs) continue to pose major risks of morbidity and mortality during yearly epidemics and periodic pandemics. The genomic instability of IAV allows it to evade adaptive immune responses developed during prior infection. Of particular concern are pandemics which result from wholesale incorporation of viral genome sections from animal sources. These pandemic strains are radically different from circulating human strains and pose great risk for the human population. For these reasons, innate immunity plays a strong role in the initial containment of IAV infection. Soluble inhibitors present in respiratory lining fluids and blood provide a level of early protection against IAV. In general, these inhibitors act by binding to the viral hemagglutinin (HA). Surfactant protein D (SP-D) and mannose-binding lectin (MBL) attach to mannosylated glycans on the HA in a calcium dependent manner. In contrast, surfactant protein A, ficolins, and other inhibitors present sialic acid rich ligands to which the HA can bind. Among these inhibitors, SP-D seems to be the most potent due to its specific mode of binding to viral carbohydrates and its ability to strongly aggregate viral particles. We have studied specific properties of the N-terminal and collagen domain of SP-D that enable formation of highly multimerized molecules and cooperative binding among the multiple trimeric lectin domains in the protein. In addition, we have studied in depth the lectin activity of SP-D through expression of isolated lectin domains and targeted mutations of the SP-D lectin binding site. Through modifying specific residues around the saccharide binding pocket, antiviral activity of isolated lectin domains of SP-D can be markedly increased for seasonal strains of IAV. Wild-type SP-D causes little inhibition of pandemic IAV, but mutated versions of SP-D were able to inhibit pandemic IAV through enhanced binding to the reduced number of mannosylated glycans present on the HA of these strains. Through collaborative studies involving crystallography of isolated lectin domains of SP-D, glycomics analysis of the HA, and molecular modeling, the mechanism of binding of wild type and mutant forms of SP-D have been determined. These studies could guide investigation of the interactions of SP-D with other pathogens.

## Introduction

Innate immunity plays a key, and surprising complex, role in host defense against influenza A virus (IAV) infection (1). The innate response is especially important in the first days after viral infection with a new strain of IAV. The response includes both soluble and cellular factors, some of which are constitutively present in the respiratory tract and others of which are elicited in response to infection. The main target cells for IAV infection are respiratory tract lining cells. Soluble innate factors intrinsically present in respiratory lining fluids have the ability to limit attachment of the virus to respiratory epithelium and thus, reduce the infectious process at the outset. Once the virus infects epithelial cells, Type 1 interferons are elicited, and these can potently limit viral infection in a non-inflammatory manner. Of interest, the virus has encoded the NS1 protein which counteracts type 1 interferon at multiple levels, and viruses lacking this protein have marked loss of fitness and infectivity (2–5). If these responses fail to adequately contain the virus, then a sometimes profound pro-inflammatory response occur consisting of cytokines and recruitment of innate immune cells including neutrophils, monocytes, and other cells. This level of response can be protective but may also lead to inflammatory injury in some instances.

Influenza A virus mainly occurs in epidemics and pandemics, either of which can cause substantial morbidity and mortality (6, 7). The current influenza season (2017–2018) has shown a sharp increase in incidence and hospitalization due to influenza, in part due to an overall vaccine effectiveness of 36% (8). Pandemics are of great concern since they reflect more marked shift in viral antigenicity and an even greater challenge for the innate immune system. Since the majority of people recover from IAV uneventfully, it is very important to identify why some develop much more severe disease. It is likely that variations in innate immunity will turn out to explain the particular susceptibility of some subjects to severe outcomes like viral pneumonia, bacterial superinfection, or cardiovascular events. Comorbid illnesses like various lung diseases, diabetes mellitus, and immune deficiencies constitute one subset of patients susceptible to severe IAV. Young children and older adults also show increased susceptibility to severe IAV, but this can vary depending on the viral strain. As an example, older adults were relatively protected from mortality during the 1918 and 2009 pandemics due to immunity acquired to remote prior exposure to similar strains (9, 10). Pandemics have been of particular concern with regard to causing mortality in young, otherwise healthy individuals (11), although the 2017–2018 seasonal epidemic already caused 63 confirmed deaths in children by early February (8).

This review will focus on the soluble factors in respiratory lining fluids that contribute to initial viral containment. The respiratory tract is the principle battle field during influenza, and it is remarkable that a virus which remains confined to the respiratory tract is able to cause such substantial morbidity and mortality. In fact, the majority of deaths caused by influenza are not the result of viral pneumonia *per se* but rather bacterial superinfection due to virus mediated blunting of antibacterial immunity (12). When IAV causes profound systemic inflammation, this may account for cardiovascular deaths during epidemics (13). For the infected host, the optimal outcome of the initial innate response includes limiting viral replication while also blunting potential excess inflammation. Several innate inhibitors, prominently including surfactant protein D (SP-D) and SP-A, mediate both of these desired activities.

## The Role of Soluble Inhibitors in Airway Lining Fluid in the Initial Defense Against IAV

We and others have characterized a variety of IAV inhibitors present in respiratory lining fluid, including SP-D, surfactant protein A (SP-A), mannose-binding lectin (MBL), H-ficolin, LL-37, and other anti-microbial peptides (14). SP-D, SP-A, and H-ficolin are constitutively present in bronchoalveolar lavage fluid (BALF). In mice, SP-D levels were shown to increase in response to IAV infection, whereas SP-A did not (15, 16). LL-37 is mainly expressed in the lung during infection or inflammation (17, 18). Whether levels of H-ficolin increase post-IAV infection has not been studied, but it is present at sufficient quantities human resting human BALF to partially reduce IAV infectivity (19). The soluble inhibitors bind to IAV and limit infectivity by different mechanisms. SP-D and MBL have been shown to bind to high mannose oligosaccharides on the viral hemagglutinin (HA) and reduce viral uptake into epithelial cells (20–23). This mechanism has been referred to as β-inhibition of IAV. A variety of other inhibitors, including SP-A, H-ficolin, pentraxins, gp-340, and mucins contain sialic acid rich attachments on their surface to which the viral HA can bind limiting the ability of the virus to reach and attach to cellular sialic acid receptors (19, 23–28). This mechanism has been termed γ-inhibition. In general, the activity of γ-inhibitors is limited somewhat by the ability of the viral neuraminidase to free the virus from attachment to them. This is evidenced by potentiation of γ-inhibitor activity by the neuraminidase inhibitor oseltamivir (25, 29, 30). This effect of most dramatic for mucins since the virus rapidly escapes attachment to these, but also evident to a lesser extent with SP-A and H-ficolin. LL-37 has a different mechanism of action from either SP-D or SP-A, which does not involve inhibition of viral HA attachment to cell (31). LL-37-treated virus is still taken up by epithelial cells but replication is limited in the early stages of the intracellular life cycle of the virus. The collectins and H-ficolin strongly induce viral aggregation, which appears to be one important of their antiviral activity. This is especially true for SP-D. LL-37, in contrast, does not induce viral aggregation.

The key role of SP-D and SP-A in limiting IAV replication and associated inflammation has been confirmed in many *in vivo* studies in mice (15, 16, 22, 32–34). Human BALF from healthy donors has strong inhibitory activity for seasonal strains and removal of SP-D significantly reduces this activity (23, 35). H-ficolin is closely related to the MBL in structure and like MBL can fix complement directly. Ficolins are abundant in blood as well but H-ficolin is present in BALF of healthy volunteer donors and removal of H-ficolin from human BALF or serum reduces antiviral activity for IAV (19). The IAV neutralizing activity of SP-D greatly exceeds that of SP-A or Ficolins (e.g., for SP-D 50% inhibition occurs at ≈10 ng/ml vs ≈10 μg/ml for SP-A or ficolins) suggesting that the mechanism of action of SP-D is more effective. This is in agreement with murine studies in which absence of SP-D has a greater impact on viral replication and inflammation than absence of SP-A (15, 16, 33, 34).

An important limitation of the action of human or rodent SP-D vs IAV is that it depends on presence high mannose oligosaccharides relatively near the sialic acid binding site of the HA. This appears to account for the relative inability of SP-D or MBL to inhibit pandemic strains IAV strains from 1918 (H1N1), 1957 (H2N2), 1968 (H3N2) and 2009 (H1N1), and avian strains like H5N1 (36). These strains have a relative lack of glycosylation their HA, especially in the region close to the sialic acid binding site (32, 37–39). H1 and H3 that cause seasonal epidemics have evolved over time within the human populations to express more glycans on the binding head of their HA and as a result are more strongly inhibited by SP-D or MBL (32, 37, 40). It has been shown that such glycans protect this critical region of the HA from recognition by antiviral antibodies. Apparently this evolutionary pressure is not as strong in avian or porcine hosts. There are actually 16 different HA subtypes most of which occur in animal reservoirs (especially birds). Most of these are resistant to inhibition by SP-D (41). Apart from the H1, H2, and H3 subtypes none of the other subtypes have established themselves in a sustained way in the human population. The H5, H7, and H9 subtypes have infected humans with relatively high mortality rates. These are, therefore, of particular concern, but fortunately they have not established sustained transmission from human to human thus far. Overall, it seems likely that the resistance of human pandemic and avian strains to SP-D may be one factor in their pathogenicity for humans, although it is clear that other properties of the HA or other genes are involved in pathogenicity as well (41).

Factors which counteract the action of SP-D, like elevated glucose in diabetes mellitus, could account for increased susceptibility to IAV as demonstrated in mice (42). Glucose, like mannose, is a competitive inhibitor of SP-D binding, although polymers of these saccharides are more potent as inhibitors. Some subjects of known susceptibility to IAV including tobacco smokers, and individuals with COPD or cystic fibrosis have reduced levels of SP-D and SP-A (43–45). In many inflammatory states, multimerization and function of SP-D are altered. Furthermore, there are polymorphic forms of SP-D and SP-A, some of which have reduced activity against IAV *in vitro* and are associated with respiratory infections (46–48). A recent study showed that some SP-A gene variants were associated with severe respiratory insufficiency in humans after infection with the 2009 pandemic H1N1 infection (49). Similar studies are needed with respect to SP-D gene variants. Reduced MBL levels due to specific polymorphisms of the MBL gene appear to increase risk for severe IAV infection as well (50).

## Determining the Molecular Mechanisms of Antiviral Activity of SP-D for Influenza

Based on these various findings summarized above which indicate the importance of SP-D in host defense against IAV, we have sought to determine the molecular basis of binding of SP-D to the IAV HA and if possible to increase activity through targeted mutations of the SP-D molecule (14). We have studied extensively two main molecular features of SP-D that determine its increased antiviral potency compared to other soluble inhibitors. These features are: (a) its multimerization property which increases its binding affinity and allows it to cause massive aggregation of viruses, bacteria, and other pathogens and (b) its lectin property which allows high affinity binding to mannosylated glycans found on many viruses and other pathogens. A variety of recombinant forms of SP-D were used in our studies as listed in Table 1.

**Table 1:** Modifications of SP-D found to increase or broaden antiviral activity for IAV.

SP-D preparation	Modification	Effect on antiviral activity
Full length recombinant human SP-D	Isolation of high molecular weight multimers	Increased activity compared to trimers or dodecamers (57, 70)
Full length chimeric protein	Rat SP-D and conglutinin (NCRD) chimera	Increased activity compared with rat SP-D (69)
Full length chimeric protein	Human SP-D and mannose-binding lectin (NCRD) chimera	Increased activity compared with similarly multimerized human SP-D (68)
NCRD trimer of human SP-D	D325A or D325S mutation	Slight increase in activity compared to wild-type NCRD (76)
NCRD trimer of human SP-D	R343V mutation	Strong increased activity compared with wild-type NCRD (75)
NCRD trimer of human SP-D	D325A(or S) combined with R343V mutation	Increased activity compared to R343V or D325A alone; significant activity vs pandemic H1N1 and H3N2 (77)

*IAV, influenza A virus; SP-D, surfactant protein D; NCRD, neck and carbohydrate recognition domain*.

### Marked Viral and Bacterial Aggregating Activity Conferred by the N-Terminal and Collagen Domains of SP-D

**Figure 1:**
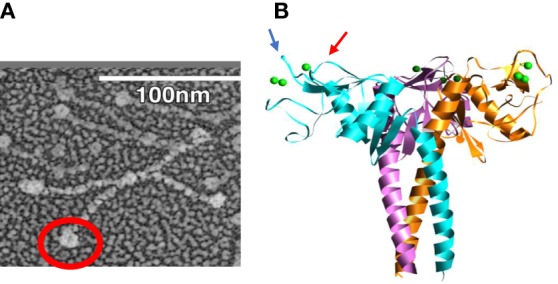
Electron micrograph of dodecameric, intact surfactant protein D (SP-D) molecule **(A)**, electron micrograph of full length dodecameric SP-D **(B)** and structure of H3N2 influenza A viruses hemagglutinin with glycan attachments. The red circle in panel **(A)** is included to highlight the globular NCRD component of the molecule. In panel **(B)**, the blue and red arrows indicate ridges surrounding the lectin binding site of the NCRD. The ridge indicated by the blue arrow is formed by the D325 residue and that shown by the red arrow is formed by R343 [this figure was adapted from Ref. (14)].

The multimerization property of SP-D is determined by its N-terminal and collagen domains, which allow for assembly into molecules with from 4 to approximately 32 trimeric arms (51). The extended nature of the collagen domain allows also for very wide separation of the trimeric heads as illustrated in Figure 1A. This structure allows for extensive cross-linking and aggregation of bacteria or viral particles which exceeds that of SP-A or MBL which have more compact structures (52, 53). The ability of SP-D to aggregate IAV is related not only to its antiviral activity but also to its ability to promote uptake of the virus or bacteria by phagocytes (52–55). The collagen domain *per se* was not found to be critical for increasing viral uptake by neutrophils or monocytes as long as the molecule can form multimers (56, 57). The neck and carbohydrate recognition domains (NCRDs) of collectins can be expressed as small separate trimers as illustrated in Figure 1B. A striking finding was that the NCRD of rodent or human SP-D nearly completely lack antiviral activity (58, 59). This finding strongly indicates the importance of cooperativity between binding heads of SP-D in effective viral binding and aggregation. This was further confirmed by showing that antibodies or other treatments which can crosslink NCRD heads without blocking the lectin binding site restore antiviral, aggregating, and opsonizing activity (58, 60, 61). A common polymorphic variant of SP-D (the Thr/Thr11 form) assembles predominantly as trimers and has greatly reduced antiviral activity *in vitro* (62). It is tempting to speculate that individuals with this polymorphism will have less ability to limit IAV infection *in vivo*.

### Distinctive Properties of NCRDs of Serum Collectins as Compared to SP-D

We found that NCRDs of MBL and a variety of serum collectins found in bovines including conglutinin, CL-43, and CL-46 have some distinctive properties which give them intrinsically greater antiviral activity (63–66). Given the strong viral aggregating properties of the N-terminal and collagen domains of SP-D, we speculated that combining this with the NCRDs of serum collectins would result in significantly potentiated antiviral activity. This was confirmed by creating chimeric molecules with the SP-D N-terminal and collagen domains and NCRDs or either conglutinin or MBL (67–69). These molecules had increased antiviral, viral aggregating, and viral opsonizing activity than either SP-D, MBL, or conglutinin. One challenge in these comparisons is that there is a great deal of variation in the extent of multimerization that occurs with molecules containing the N-terminal and collagen domains of SP-D. The very high molecular weight multimers of human SP-D occur both naturally and in recombinant preparations and have increased antiviral activity compared to dodecamer forms that are composed of four trimeric arms (53, 70). Similar high molecular weight forms are observed with porcine SP-D (71).

### Engineering the NCRD of SP-D to Resemble Those of Serum Collectins

When comparing SP-D and serum collectins, key differences are evident in residues adjacent to the lectin binding site calcium. We speculated that these differences could account for increased viral binding ability of serum collectin NCRDs. CL-43 has a three amino acid insertion of Arg-Ala-Lys (RAK) adjacent to the conserved EPN sequence, which is at the base of the saccharide binding pocket. Addition of this insertion into the NCRD of SP-D conferred increased affinity for mannan and increased ability to inhibit IAV as well (60, 61, 65, 66, 72). Further insights were obtained from study of the crystal structure of the SP-D NCRD (73, 74). Two residues, aspartic acid 325 (D325) and arginine 343 (R343) of SP-D, account for ridges on either side of the lectin binding site as illustrated in Figure 1B. R343 is replaced by highly hydrophobic residues (valine or isoleucine) in the serum collectins. Simple substitution of one of these residues (or to a lesser extent alanine) for R343 caused a marked shift in preference of binding to specific oligosaccharides and also marked increase in viral binding and inhibition (75). In contrast, an R343K substitution did not alter antiviral of glycan binding activity.

The D325 residue also differed in some serum collectins (e.g., there is a serine in there in conglutinin), and replacement of D325 with an alanine or serine caused modest increase in antiviral activity (much less than the R343V change) (76, 77). Even the slight D325N alteration (to mimic rat or mouse SP-D) led to a slight shift in saccharide and viral binding (78). More importantly the combined change of D325A(or S) along with R343V in the NCRD of human SP-D enabled greater antiviral activity than either substitution alone (76, 79). The D325A + R343V NCRD had neutralizing activity comparable to full length SP-D dodecamers for seasonal IAV strains and also caused marked viral aggregation despite absence of the N-terminal and collagen domains (77, 80). In addition, administration of D325A + R343V to mice along with an SP-D sensitive (but pathogenic) viral strain significantly reduced viral loads and mortality, whereas the wild-type SP-D NCRD did not (77). A striking additional finding was that the D325A + R343V NCRDs or the related molecule D325S + R343V were able to inhibit pandemic H1N1 of 2009 or 1918 *in vitro* (76). Neither of these strains are inhibited by full length SP-D nor wild-type NCRD. In addition, these double mutant molecules had greater ability to inhibit the 1968 H3N2 pandemic strain than full length SP-D. The D325S + R343V NCRD also reduced viral loads and mortality in mice infected with this pandemic H3N2 strain while with type NCRD did not (76).

### Molecular Basis of Increased Neutralization and Viral Aggregation by Double Mutant NCRDs

**Figure 2:**
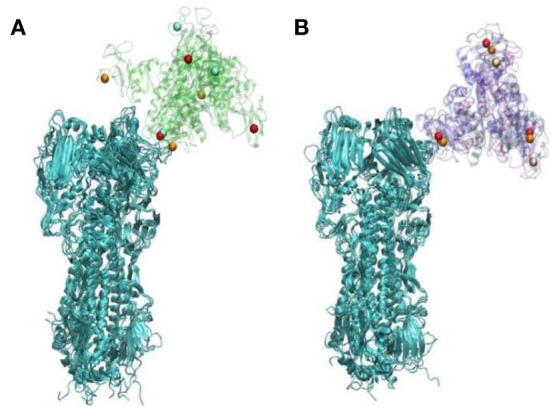
Differences in orientation in hemagglutinin (HA) complexes with WT **(A)** or D325A + R343V **(B)**—this figure represents the endpoints of three repeated simulations. Calcium ions are represented in orange, red, and white; WT and D325A + R343V are in transparent cartoon representation in green and purple, respectively; HA is shown in cartoon representation in cyan [this figure was reprinted with permission from Goh et al. (79)].

X-ray crystallographic studies by Drs. Barbara Seaton and Michael Rynkiewicz (Boston University School of Medicine, Physiology and Biophysics) have shown how man9 (an extended mannose chain comparable to those found on viral high mannose glycans) binds to wild-type human SP-D NCRD and the D325A + R343V mutant NCRD (79). They were able to show that mannose chains assume a different orientation with respect to the NCRD surface on the mutant form. This was accounted for by the fact that in the mutant NCRD the penultimate mannose in the chain binds in the lectin site. This contrasts with the wild-type NCRD in which the terminal mannose binds. This finding correlated with increased binding affinity of the mutant NCRD to extended mannose chains as assessed by glycan array studies and increased binding to the Phillipines 82 H3N2 (Phil82) seasonal viral HA by surface plasmon resonance studies (79). Prior seminal studies by Patrick Reading and Margot Anders had shown the importance of the glycan at position 165 for binding of SP-D or MBL (20–22). Dr. Joseph Zaia (Boston University School of Medicine, Biochemistry) and colleagues were able to confirm that this glycan is of the high mannose type. Based on these findings and those of Drs. Seaton and Rynkiewicz, Drs. Boon Guh, and Klaus Schulten (University of Illinois, Urbana-Champaign, Physics) were able use molecular dynamics to show that the mode of binding of the mutant NCRD to the Phil82 H3N2 HA, using a high mannose glycan at position 165 as the binding site (79). These studies revealed two important insights. Given the different mode of binding of the mutant NCRD to the glycan (i.e., to the penultimate mannose in the chain) it was: (1) able to more fully cover-up the sialic acid binding site of the HA as compared to the wild-type NCRD and (2) able to bind in such a way that the other two lectin sites on the trimer remains exposed to such that they could attach to other HA molecules (see Figure 2). The latter observation provides a potential explanation for viral aggregation mediated by the mutant NCRD (in contrast to wild-type NCRD).

**Figure 3:**
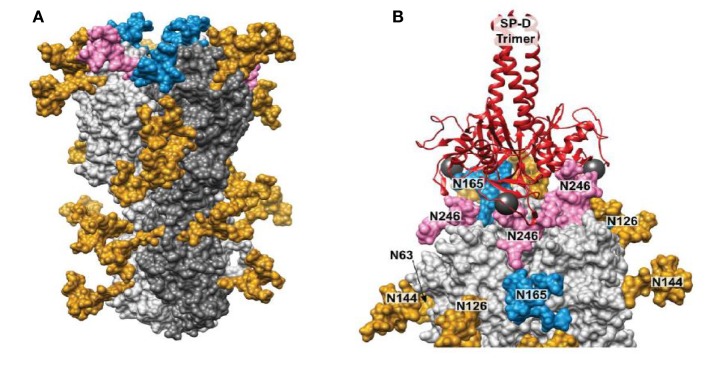
Structure modeling of influenza A virus (IAV) hemagglutinin (HA) and molecular dynamics result showing binding of surfactant protein A (SP-D) neck and carbohydrate recognition domain trimer to the HA. **(A)** 3D model of glycosylated Phil82 hemagglutinin trimer (HA0). One HA monomer is shown in dark gray. Glycans at Asn165 and Asn246 are shown in blue and pink, respectively. **(B)** A model of trimeric SP-D (red ribbons) bound to three N-glycans on the H3 head domain (light gray surface). The 3D structure of the glycosylated H3 was taken from the MD simulation. In this snapshot, Manα residues at sites Asn 246, Asn 165, and Asn 246 on different protomers were approximately 45 Å apart, such that SP-D could bind each residue simultaneously. The calcium atoms found in each SP-D Manα binding site are shown (dark gray spheres) [this figure adapted from Khatri et al. (81) through free usage *via* Creative Commons CC-BY license].

Drs. Kshitij Khatri and Joseph Zaia have further expanded on these studies by use of integrated omics and computational biology to determine the true site occupancy and actual glycoforms present on the seasonal Phil82 HA and its bovine serum resistant variant, Phil82/BS. Of interest, not all potential glycan attachment sites on the parental predicted from the protein sequence of the parental Phil82 strain were in fact glycosylated. This supports the importance of actual determination of the location and type of glycan attachments on viral envelope proteins as opposed to basing observations only on attachments predicted by the protein sequence. The structure of the glycosylated HA trimer is shown in Figure 3A. Furthermore, the Phil82/BS strain was shown to lack two high mannose glycans on the exposed tip of the HA (those at position 165 and 246).

Using molecular dynamic studies Drs. Khatri and Zaia found that the distance between SP-D NCRD trimeric heads is compatible with binding of all three heads to a single HA molecule (also trimeric) through various potential interactions with glycans at 165 and 246. An interaction of this kind is shown if Figure 3B. This provides additional explanation for the ability of SP-D to strongly inhibit certain seasonal H3N2 strains. Note that the pandemic H3N2 strain of 1968 had the glycan at 165 but lacked several other potential glycan attachment sites on its HA. This may account for the reduced ability of wild-type SP-D to inhibit this strain as compared to more recent H3N2 strains including Phil82. Presumably, a similar result may obtain with seasonal vs pandemic H1N1 strains. Note that the 1918 and 2009 H1N1 pandemic strains both have a glycan attachment site at position 104. This attachment site has also been predicted to be important for attachment of collectins (20). If this attachment is high mannose as expected, why then are the pandemic H1N1 strains not inhibited by collectins? It is likely that other high mannose glycans (or one other glycan) must be present on seasonal H1N1 strains that developed between 1977 and 2009 during human circulation provide a second important attachment site for collectin. Future studies will hopefully answer this question.

Another very important line of investigation carried out by Drs. Martin van Eijk and Henk Haagsman (University of Utrecht, Netherlands) has shown that porcine SP-D has increased ability to inhibit seasonal IAV strains but also a number of pandemic and avian strains (82, 83). The importance of studying porcine SP-D is that pigs are a source through which pandemic strains can be ultimately transmitted to humans and in the case of the 2009 H1N1. Pigs appear to provide a vessel through which human, porcine, and avian strains can become reassorted to generate new strains potentially adaptable to human transmission. How then does this relate to the increased antiviral activity of porcine SP-D? One speculation is that pigs have partial protection through conferred by innate inhibitors like SP-D allowing them to be infected with multiple IAV strains without strong manifestations of illness. In any case, study of porcine SP-D has revealed important insights into mechanism of collectin inhibition of IAV. Of great interest, porcine SP-D is the only known form of the molecule that has an N-linked glycosylation site on its NCRD. This site has been shown to be highly sialated and to contribute to the enhanced antiviral activity of porcine SP-D (82–86). The presence of the N-linked sugar gives porcine SP-D a dual mechanism of action in which it can bind to viral glycans while also presenting a decoy sialic acid rich ligand to which the viral HA binds. Initial studies by Dr. van Eijk also showed that key residues on the binding surface of the porcine SP-D carbohydrate recognition domain (CRD) differ compared to human or rodent SP-D (71). As in the case of serum collectins, these changes allow the porcine NCRD (even without the N-linked glycan) to inhibit IAV to a much greater extent than wild-type human or rat SP-D NCRDs (87). Further studies of porcine SP-D are important especially given its ability to inhibit pandemic and avian IAV strains.

## Future Directions

There are other potential impacts of HA glycans including their ability to shield regions of the HA from antibody recognition (9, 88, 89), alteration of the binding properties of the HA (90), and effects on interactions with other host defense lectins like the macrophage mannose receptor, DC-SIGN, or galectins all of which play roles in host defense against IAV (37, 91, 92). It should be noted that such lectin interactions may not always lead to viral inhibition, but can also provide an alternate route for viral entry into and infection of cells (91, 93). Future studies to determine molecular mechanisms of binding to these other host defense lectins will be important.

An important question that is not fully resolved is how SP-D (or SP-A) blunts inflammatory reactions during IAV infection. This immunomodulatory effect is likely to be an important aspect of their protective effect *in vivo*. One hypothesis is that SP-D reduces inflammation simply by reducing viral replication. Alternatively, SP-D could independently act on inflammatory cells to limit their activity. We recently found that SP-D inhibits replication of seasonal IAV in human monocytes and reduces monocyte tumor necrosis factor (TNF) responses to the virus (56). In that study, reduction of the TNF response could be demonstrated even when viral replication was not reduced. Further studies are warranted to determine potential independent effects of SP-D or SP-A on IAV induced lung inflammation. It should be noted that elevated serum levels of SP-D have been associated with worse outcome of severe H1N1 IAV infection (94). Serum levels of SP-D are elevated in a number of conditions in which there is severe lung inflammation and may reflect breakdown in alveolar capillary barriers (43, 95), rather than some adverse effect of SP-D *per se*.

Finally, therapeutics based on SP-A and SP-D can considered with respect to IAV or other respiratory viruses. Potential challenges include selection of patients for such treatment and how to administer protein-based treatments into the airway. Another option is trying to increase endogenous SP-D production as a therapeutic maneuver. It is possible that steroid treatment which is beneficial in some lung infections (e.g., *Pneumocystis carinii*) acts in part through this mechanism. GM-CSF increases surfactant protein production and contributes to host defense against IAV in mice (96, 97). We collaborated with Dr. Frank McCormack of University of Cincinnati School of Medicine to see if keratinocyte growth factor (KGF) would improve outcome of IAV infection in mice based on its known ability to increase pulmonary collectins. However, KGF treatment unexpectedly increased viral replication and mortality in IAV infected mice through a mechanism involving the ability to KGF to increase alveolar epithelial cell proliferation (98). In any case, further research on the role of SP-D in human IAV infection and efforts to harness its antiviral activity for therapeutic purposes remain priorities given the major, ongoing health impacts of IAV and limited options for treatment.

## Conclusion

Surfactant protein D appears to play an important role in limiting IAV replication and blunting inflammatory responses to the virus. In this review, we detail findings which demonstrate the molecular mechanism of binding to the viral HA to inhibit infectivity and also the mechanism through which SP-D can induce viral aggregation. Aggregation of viruses and other pathogens may be a major mechanism through which SP-D contributes to first line host defense in the airway. We also review, how small changes in the SP-D NCRD are able to increase binding to high mannose glycans and inhibition of IAV. The relative paucity of glycans on the HA of pandemic and avian IAV strains accounts for failure of SP-D to inhibit these strains and this in turn may be one factor in the high level of pathogenicity of these strains. To some extent, modification of the NCRD of SP-D enables it to inhibit some pandemic IAV strains, and this may have therapeutic relevance. Study of molecular interactions of SP-D with IAV has provided a very detailed look at how a lectin-based innate immune protein can mediate binding and defense against a pathogen. Similar studies could be carried out with other pathogens or host defense lectins.

## Author Contributions

KH supervised much of the work summarized in this review as well doing writing and review of the manuscript. I-NH, XL, and MW carried out experiments described in the review but also wrote and edited parts of the manuscript. All authors approved the final version of the manuscript.

## Conflict of Interest Statement

The authors declare that the research was conducted in the absence of any commercial or financial relationships that could be construed as a potential conflict of interest.
